# The year of the tiger and the year of tiger genomes!

**DOI:** 10.1111/1755-0998.13726

**Published:** 2022-11-06

**Authors:** Anubhab Khan

**Affiliations:** ^1^ SBOHVM University of Glasgow Glasgow UK; ^2^ Department of Biology Pennsylvania State University University Park Pennsylvania USA; ^3^ National Centre for Biological Sciences, TIFR Bangalore India

## Abstract

Tigers are endangered apex predators. They typify endangered species because they are elusive, rare, and face numerous threats across their range. Tigers also symbolize conservation. However, it is very difficult to study tigers because of their stated nature. Also, tiger conservation is a geopolitically sensitive topic, making it difficult to use the studies to propose evidence‐based management that allows their recovery, especially in the context of conservation genetics. Zhang et al. (*Mol. Ecol. Resour.*, 2022) have created very valuable and rare resources to aid the community in conserving tigers. First, they present chromosome level genome assemblies of a South China tiger and an Amur tiger. Second, they present whole genome sequences of 16 captive South China tigers. Additionally, by using the assemblies they model the demographic history of these populations, estimate inbreeding and the potential threats they face in captivity. This approach is particularly important because genetic management is now the only remaining option for South China tigers, because they are extinct in the wild. In other words, captive individuals are our only hope for some day restoring the wild populations of South China tigers.

The genome assemblies presented by Zhang et al. (2022) are some of the best tiger assemblies available so far and comparable even to the domestic cat reference genomes. Three out of six extant tiger subspecies account for a total of six chromosome‐level assemblies (two South China tigers [Wang et al., 2021; Zhang et al., 2022], two Amur tigers [Zhang et al., 2022, dnazoo.org], two Bengal tigers [Shukla et al., 2022]). Malayan tigers also have a decent genome assembly (Armstrong et al., 2021). Taken together, these assemblies have already started contributing to understanding not only the evolution of tigers (Armstrong et al., 2021; Khan et al., 2021; Sagar et al., 2021; Shukla et al., 2022; Zhang et al., 2022) but also how these populations can be sustained into the future (Khan et al., 2021; Zhang et al., 2022). They allow scientists to develop tools for tiger conservation and management (Khan et al., 2022, 2020; Natesh et al., 2019; DNAzoo.org). Such data sets are rare for Asian species, especially published by Asian laboratories (Khan & Tyagi, 2021) giving hope that more endangered species in Asia will have their genome assembled.

Amazingly, there are already approximately 170 whole genomes of tigers sequenced at varying depth (Armstrong et al., 2021; Khan et al., 2021; Liu et al., 2018; Sagar et al., 2021), but only four whole genomes from South China tigers available in the SRA database of NCBI (https://www.ncbi.nlm.nih.gov/sra; SRR7651468, SRR7651469, SRR7651471, SRR7152387). The 16 additional South China tiger genomes presented in Zhang et al. (2022) are extremely valuable resources in the study of tigers because South China tigers are a basal lineage of tigers (Liu et al., 2018). Interestingly, these genomes also shed light on divergence times between tiger subspecies. Demographic models with these data support a recent divergence (~7000 years, this study, also suggested in Armstrong et al., 2021) compared with an older divergence (~65,000–100,000 as suggested by Liu et al., 2018). Additionally, Zhang et al. (2022) observe that the populations of Amur and South‐china tiger subspecies had been declining until 5000 years ago, after which the South China tiger population declined sharply. It is possible that in recent timescales (10,000–800 years ago), tiger populations could have become fragmentated and multiple South China tiger subpopulations could have existed (Wang et al., 2021) just like there were multiple Bengal tiger populations in this time period (Armstrong et al., 2021). Signals of structured populations may be indistinguishable from that of changes in population size (Mazet et al., 2015). It is important for future studies to use historic samples of tigers to understand the evolution and demographic history of tigers; and to bolster the claims about the splitting times of the various subspecies.

Genome assemblies of the quality presented in Zhang et al. (2022) are essential for accurate estimates of inbreeding using runs of homozygosity (ROH). Fragmented genome assemblies are unable to detect long ROH reliably (Shukla et al., 2022). It is because of the high‐quality assembly Zhang et al. (2022) detected ROH segments, spanning approximately 66% of a chromosome, that were shared across the south china tigers. The genomic signatures of inbreeding in captive South China tigers were found to be more prevalent than in captive Amur tigers (Zhang et al., 2022). This signature is probably caused by historic bottlenecks and strong founder events that the South China tiger populations might have faced in addition to the recent inbreeding.

Zhang et al. (2022) attempt to estimate deleterious allele load using the method demonstrated in Feng et al. (2019). The method assumes that the population is at a local fitness maximum and any missense mutation that changes the ancestral amino acid to a very different one (for example, changing a hydrophilic amino acid to a hydrophobic one) must be deleterious. It is important to identify the ancestral nucleotide at the genomic loci for using this method. However, Zhang et al. (2022) do not identify the ancestral allele at the loci. Thus, we remain unsure which allele in a locus is deleterious and how many deleterious alleles are already fixed in a population. For loss of function (LOF) mutations, all stop gain, splice acceptor and donor variants were classified as deleterious. An ambiguity remains here too about the ancestral state of these alleles. However, the number and identity of loci hosting potential deleterious alleles, that are not already fixed in the population, would broadly remain unaltered by this approach. Zhang et al. (2022) identify 170 South China tiger specific mutations that were in genes responsible for reproduction, growth and development. This in conjunction with the observation that South China tigers face lower fecundity, makes the loci detected in the study very important for the community. Detection of causal mutations for disease is rare for wild animals, thus having the identities of the candidate loci for further verification is very helpful. A thorough investigation affirming that the candidate mutations detected in the study, are causal for unhealthy sperms, foetal abortions and abnormal tissue and organ development, as listed in Zhang et al. (2022), would present several avenues for tiger conservation. There is potential for these mutations to be monitored in the wild using SNP panels (for example, Khan et al., 2022; Natesh et al., 2019) and avoiding these mutations in the captive breeding facilities. Potential genetic engineering of an ancestral allele at these loci may be important for genetic rescue, especially in captivity as the study shows that the South China tiger population might need genetic rescue. Previously, genome‐wide *F*_ST_ between populations has been suggested as an effective indicator of genetic rescue from high frequency deleterious alleles (Khan et al., 2021). With the data set presented in Zhang et al. (2022) and the existing whole genome data sets, it might be possible to choose a suitable population for genetic rescue. Since Amur and Sumatran tiger genomes are evolving under selection (Armstrong et al., 2021); Bengal, Malayan and Indochinese tigers might be good candidates to consider based on the population divergence.

Overall, the genome assemblies and analyses presented by Zhang et al. (2022) are vital for the continued progress of tiger research. The genome annotations of the genomes and the detection of the loci hosting potential deleterious alleles will help on‐ground conservation efforts soon. With the release of so many tiger genome sequences and assemblies (Shukla et al., 2022; Wang et al., 2021; Zhang et al., 2022), this is truly the year of the tiger genomes. What better way to celebrate the year of the tiger?

## CONFLICT OF INTEREST

There is no conflict of interest.

## Data Availability

Data sharing is not applicable to this article as no new data were created or analyzed in this study.
